# Ribosomal profiling of human endogenous retroviruses in healthy tissues

**DOI:** 10.1186/s12864-023-09909-x

**Published:** 2024-01-02

**Authors:** Nicholas Dopkins, Bhavya Singh, Stephanie Michael, Panpan Zhang, Jez L. Marston, Tongyi Fei, Manvendra Singh, Cedric Feschotte, Nicholas Collins, Matthew L. Bendall, Douglas F. Nixon

**Affiliations:** 1 Division of Infectious Diseases, Department of Medicine, Weill Cornell Medicine, New York, NY 10021 USA; 2 Department of Molecular Biology and Genetics, Cornell University, Ithaca, NY 14850 USA; 3 Clinical Neuroscience, Max Planck Institute for Multidisciplinary Sciences, City Campus, Göttingen, Germany; 4 Jill Roberts Institute for Research in Inflammatory Bowel Disease, Weill Cornell Medicine, New York, NY 10021 USA

**Keywords:** Human endogenous retrovirus (HERV), Ribosomal profiling (RiboSeq), Protein translation, Dark genome, Endoretrotranslatome (ERT)

## Abstract

Human endogenous retroviruses (HERVs) are the germline embedded proviral fragments of ancient retroviral infections that make up roughly 8% of the human genome. Our understanding of HERVs in physiology primarily surrounds their non-coding functions, while their protein coding capacity remains virtually uncharacterized. Therefore, we applied the bioinformatic pipeline “hervQuant” to high-resolution ribosomal profiling of healthy tissues to provide a comprehensive overview of translationally active HERVs. We find that HERVs account for 0.1–0.4% of all translation in distinct tissue-specific profiles. Collectively, our study further supports claims that HERVs are actively translated throughout healthy tissues to provide sequences of retroviral origin to the human proteome.

**Supplementary Information:**

The online version contains supplementary material available at 10.1186/s12864-023-09909-x.

## Background

Human endogenous retroviruses (HERVs) persist within the genome as the legacy of ancient retroviral infections that integrated into the germline [1, 2]. Germline embedded retroviruses then transmit vertically where over time they then accumulate mutations or deletions that prohibit infectious particle formation. Once a retrovirus no longer produces infectious particles, they are deemed “endogenous” [1]. Endogenization is not an instantaneous process, but instead occurs through complex transgenerational invasion of genomic sequences by a retrovirus, as demonstrated by the active endogenization events occurring in Koala species [3]. Once retrovirus has invaded the germline of a host specific, endogenization can then be driven by a multitude of factors, such as xenotropic restriction [4], mutations [1, 2], host-antiviral responses [5], and recombination events [6]. Collectively, HERVs make up about 8% of human genetic material [2, 7, 8], and have therefore substantially impacted the genome. While HERVs are mostly inactive [9] and none are replication competent like the ERVs of other mammals [10], many do display spatiotemporal activity in somatic [11–14] and developing cells [15–21] alike. Since their endogenization, many HERV elements have been coopted to accomplish molecular tasks in which are observable throughout reproduction [22, 23], immune responses [24, 25], and cell type specific transcription [11, 17, 19, 26]. Our current understanding of HERVs is primarily derived from their genomic and transcriptomic functions while little is known about their protein encoding capabilities.

Here, we performed the first large-scale characterization of HERV translation in healthy tissues by analyzing publicly available ribosomal profiling (RiboSeq) datasets [27]. RiboSeq quantifies the translatome by sequencing the short fragments (~ 25-35 bps) of ribosomal protected RNA, therefore providing a ‘snapshot’ of protein production [28]. By applying the bioinformatic pipeline ‘hervQuant’ [29] to publicly available RiboSeq data, we quantify the translational abundance of over 3000 annotated HERV proviruses [30] across an atlas of healthy tissue and cell types by aligning ribosomal protected short RNA sequencing fragments to full length proviruses. Collectively, this approach provides the first comprehensive characterization of actively translated HERV proviruses under healthy conditions. We term the collective of HERV proteins undergoing translation as the “endoretrotranslatome” (ERT) and suggest further investigation into the ERT as an understudied component of human health.

## Results and discussion

**Fig. 1:**
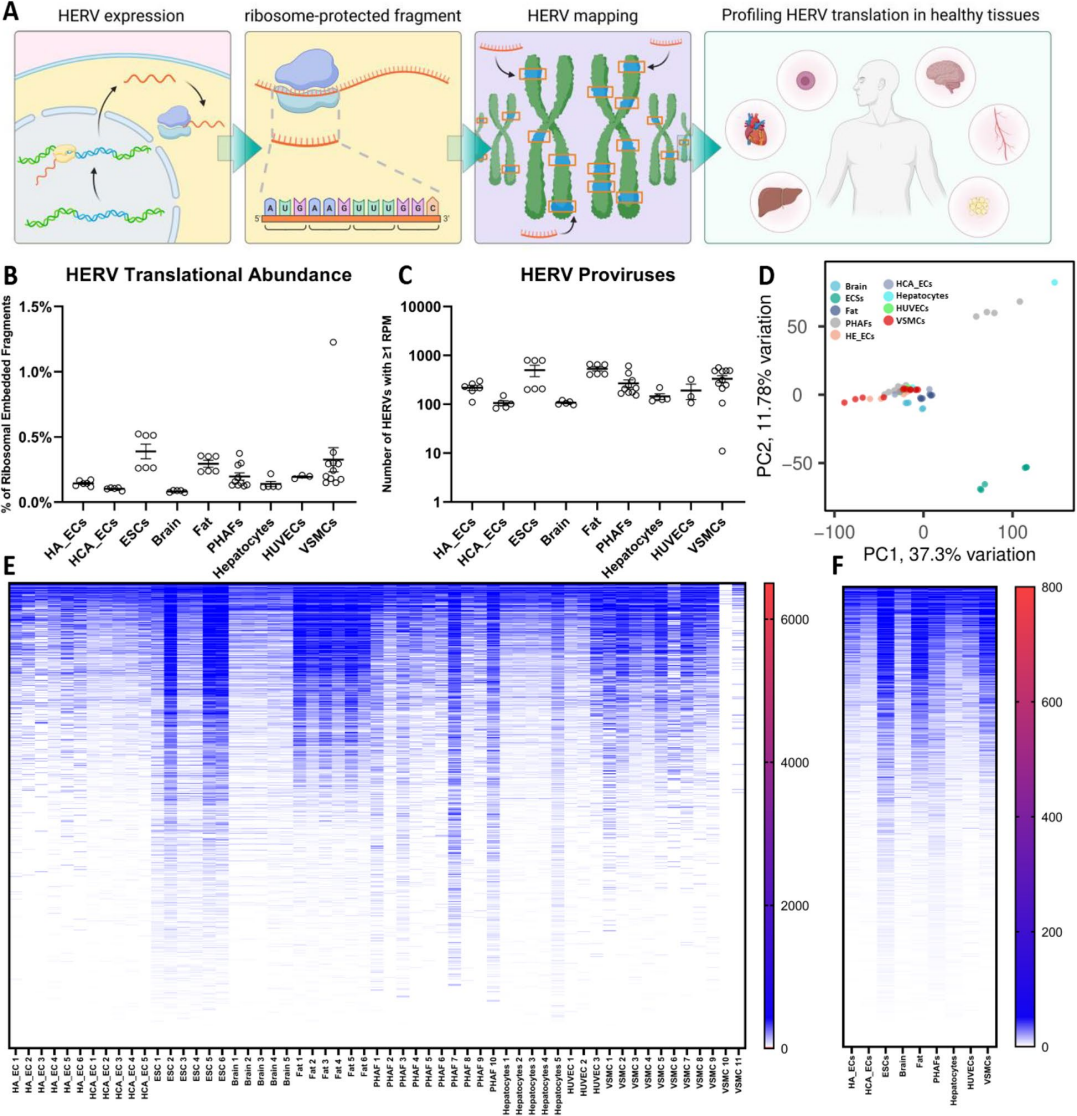
Ribosomal profiling reveals active translation of HERV proviruses in healthy tissue and cell types. **a** Schematic overview of workflow for profiling HERV proviral abundances from RiboSeq data. **b** HERV-aligned reads as a percentage of all filtered sequencing reads per sample. Dots indicate individual biological replicates with the graphed mean. Error bars indicate ± standard error of the mean (SEM). **c** Sum number of HERV proviruses possessing ≥1RPM per sample. Dots indicate individual biological replicates with the graphed mean. Error bars indicate ± SEM. **d** PCA plot of all tissue and cell types based on HERV-aligned ribosomal profiling reads alone. **e** Individual sample RPM abundances of all HERV proviruses per sample clustered per cell or tissue type. HERVs are listed in descending order by average RPM abundance. **f** Average RPM abundances of all HERV proviruses per cell or tissue type. HERVs are listed in descending order by average RPM abundance

Despite characterizations of multiple uniquely identified HERV proteins [20, 23, 31–50], HERV-derived peptides as neoantigens in cancers [29, 51–67], and the identification of open reading frames (ORFs) embedded within HERV loci [2, 30, 47, 68–79], little is known regarding whether or not HERVs are readily translated in healthy tissues specifically. For this purpose, we aligned ribosomal embedded mRNA fragments from the RiboSeq atlas [27] with an annotation of proviral sequences [30] using hervQuant [29], a biologically validated pipeline that accurately depicts HERV translation. Using this approach, we provide the first large-scale examination of the ERT in brain, liver, and fat tissues, as well as within cell types such as vascular smooth muscle cells (VSMCs), embryonic stem cells (ESCs), human aorta endothelial cells (HA_ECs), human coronary artery endothelial cells (HCA_ECs), human umbilical vein endothelial cells (HUVECs), and primary human atrial fibroblasts (PHAFs). (Fig. 1A; Table S1). We found that all samples display detectable translation of HERV products ranging from 0.08% (brain) to 0.39% (ESCs) of all translation (Fig. 1B; Table S2). We next quantified the number of HERV proviruses that contain ≥1 read per million (RPM) and found that fat tissue displayed the most diverse expression profiles with an average of 533 HERV proviruses surpassing this threshold per sample, while HCA_ECs displayed the least diverse expression profile averaging only 105 distinct HERV proviruses that surpass this threshold (Fig. 1C). Principal component analysis (PCA) plots based on HERV protein production alone demonstrate that ESCs can be distinguished from other sample types by the ERT alone, while somatic sample types are indistinguishable from one another (Fig. 1D). This ESC-specific profile is largely attributed to high translation of the HERVHF superfamily (Fig. S1; Table S3), a large HERV clade whose activity coordinates early embryonic development [16, 80]. Heatmaps of HERV proviral transcript abundances organized in descending order of RPM abundance showcase discrete changes in the ERT between tissue and cell types (Fig. 1E-F). Collectively, these data demonstrate that HERVs are translated throughout healthy tissue types with-site specific translational profiles.

**Fig. 2:**
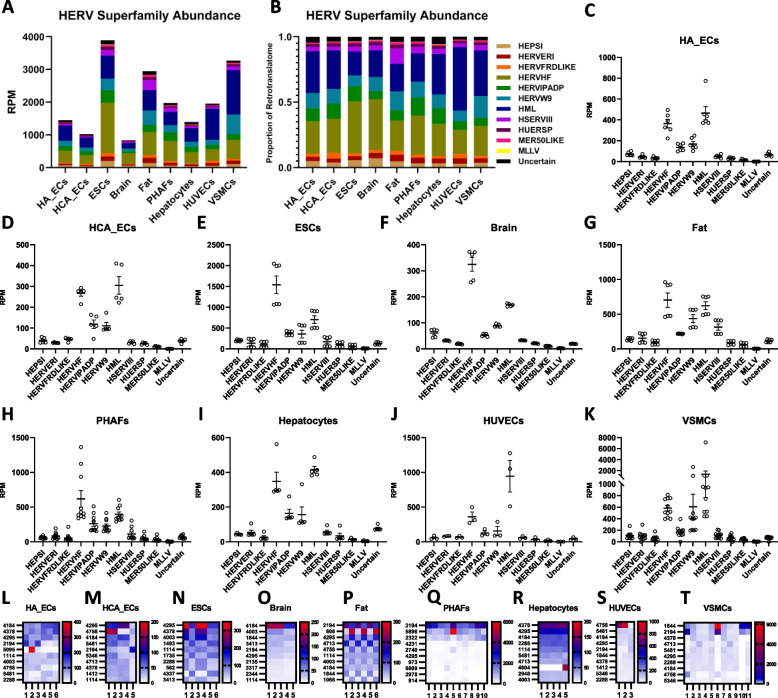
Profiling of the endoretrotranslatome. **a** HERV superfamily RPM abundance averages per tissue or cell type. RPM values are calculated based on the total number of filtered reads per sample. **b** HERV superfamily abundance averages per sample type as a proportion of all HERV-aligned reads. **c** HERV superfamily RPM abundances per sample in HA_ECs. Dots indicate individual biological replicates with the graphed mean. Error bars indicate ± SEM. (*n* = 6). **d** HERV superfamily RPM abundances per sample in HCA_ECs. Dots indicate individual biological replicates with the graphed mean. Error bars indicate ± SEM. (*n* = 5) **e** HERV superfamily RPM abundances per sample in ESCs. Dots indicate individual biological replicates with the graphed mean. Error bars indicate ± SEM. (*n* = 6) **f** HERV superfamily RPM abundances per sample in brain tissue. Dots indicate individual biological replicates with the graphed mean. Error bars indicate ± SEM. (*n* = 5) **g** HERV superfamily RPM abundances per sample in fat tissue. Dots indicate individual biological replicates with the graphed mean. Error bars indicate ± SEM. (n = 6) **h** HERV superfamily RPM abundances per sample in PHAFs. Dots indicate individual biological replicates with the graphed mean. Error bars indicate ± SEM. (*n* = 10) **i** HERV superfamily RPM abundances per sample in hepatocytes. Dots indicate individual biological replicates with the graphed mean. Error bars indicate ± SEM. (n = 5) **j** HERV superfamily RPM abundances per sample in HUVECs. Dots indicate individual biological replicates with the graphed mean. Error bars indicate ± SEM. (*n* = 3) **k** HERV superfamily RPM abundances per sample in VSMCs. Dots indicate individual biological replicates with the graphed mean. Error bars indicate ± SEM. (*n* = 11) **l** Heatmap displaying RPM abundances of the top 10 most highly translated HERVs in HA_ECs. (n = 6) **m** Heatmap displaying RPM abundances of the top 10 most highly translated HERVs in HCA_ECs. (n = 5) **n** Heatmap displaying RPM abundances of the top 10 most highly translated HERVs in ESCs. (n = 6). **o** Heatmap displaying RPM abundances of the top 10 most highly translated HERVs in brain tissue. (n = 5). **p** Heatmap displaying RPM abundances of the top 10 most highly translated HERVs in fat tissue. (n = 6). **q** Heatmap displaying RPM abundances of the top 10 most highly translated HERVs in PHAFs.(n = 10). **r** Heatmap displaying RPM abundances of the top 10 most highly translated HERVs in hepatocytes. (n = 5). **s** Heatmap displaying RPM abundances of the top 10 most highly translated HERVs in HUVECs. (n = 3). **t** Heatmap displaying RPM abundances of the top 10 most highly translated HERVs in VSMCs. (n = 11)

Next, we profiled the ERT based on phylogeny. Summary data demonstrates the average RPM (Fig. 2A) and proportional (Fig. 2B) abundances of all HERV superfamilies across tissue and cell types. Collectively, the HML (HML1 through HML10, including HML2), HERVW9, HERVIDADP, and HERVHF superfamilies were most translationally active in healthy tissues (Fig. S2). Next, analysis of RPM abundances amongst HERV superfamilies in HA_ECs (Fig. 2C), HCA_ECs (Fig. 2D), ESCs (Fig. 2E), brain (Fig. 2F), fat (Fig. 2G), PHAFs (Fig. 2H), hepatocytes (Fig. 2I), HUVECs (Fig. 2J), and VSMCs (Fig. 2K) suggests that sample type is the dominant factor in determining HERV translation, while interindividual discrepancies are a secondary determinant. Proportional abundances of HERV superfamilies further supports this conclusion (Fig. S3). Next, we identify the 10 most highly translated HERVs in HA_ECs (Fig. 2L), HCA_ECs (Fig. 2M), ESCs (Fig. 2N), brain (Fig. 2O), fat (Fig. 2P), PHAFs (Fig. 2Q), hepatocytes (Fig. 2R), HUVECs (Fig. 2S), and VSMCs (Fig. 2T) demonstrate distinct changes in translational abundances (Table S1). Proviruses HERV_4295 (Fig. S4A) and HERV_4184 (Fig. S4B) possess conserved RPM abundances, suggesting conserved roles in ubiquitous processes. Meanwhile, highly translated proviruses such as HERV_1844 (Fig. S4C), HERV_4378 (Fig. S4D), and HERV_4231 (Fig. S4E) contain differential RPM abundances and may instead contribute to specialized roles indicative of a local environment.

Our analyses demonstrate that HERV-provirus aligned reads make up a surprising portion of the human translatome, encompassing roughly between 0.1–0.4% of all translation in a site-specific manner. Unsurprisingly, the ERT displays substantial diversity across tissue sites. As the expression of HERVs at the RNA level is tightly regulated by an excessive complement of epigenetic modifications [9], their translation with little interindividual discrepancies suggests that their expression at the protein level is likely by design and not inadvertent. Post-translation, HERV protein stability and function may be rapidly compromised by the host via post translational modifications [81] or by the targeted clearance of dysfunctional protein aggregates [82, 83], and therefore a limitation of this study pertains to their unknown half-life. In example, our results find that paraneoplastic Ma antigen 1 (PNMA1), a domesticated LTR retrotransposon capsid containing a neuronal autoantigen associated with paraneoplastic neurological pathologies [84], is translated throughout all tissue types tested (Fig. S5). Therefore, going forward considering the rate of transcription, translation, and degradation would provide the most comprehensive determination of HERV activity [85].

## Conclusions

In this study, we demonstrate that HERVs, acquired via ancient retroviral infections, are translationally active elements. Previous misconceptions suggested that HERVs were merely inert or parasitic sequences, however it is now appreciated that HERVs innervate host physiology [86], regulate transcriptional networks [87, 88], contribute to the transcriptome [11–13], and provide retroviral motifs that propagate immunity [24, 25]. Here, we demonstrate that HERVs are translated in greater than anticipated proportions, and that HERV proteins are a reservoir of poorly defined macromolecules that may impact human health and disease. Previous studies have shown that a diverse profile of HERVs are expressed that the RNA level throughout various tissue sites, and that HERV RNAs make up roughly 0.19–1.91% of all polyadenylated RNA in site-specific manners [12]. Additionally, the authors demonstrate HERV RNA activity is sensitive to confounding variables, such as background and age [12]. Transcriptional activity of the HML and HERHF superfamilies, which we found to be most abundant in the ERT, has previously been detected in fully differentiated somatic tissues [12, 13, 89, 90]. Additionally, in ESCs many HERV elements are derepressed, and HERVH elements are highly active and contribute to cellular ESC cell specific processes [16, 91]. Therefore, it is unsurprising that we see the highest proportions of HERV translation globally and from the HERVHF family in ESCs.

In accordance with previous observations of HERV activity in the transcriptome and genome, we now demonstrate that HERV RNAs can be found in the ribosome of healthy human tissues. While ribosomal RNA content does not perfectly equate to stable protein levels, as demonstrated by the translational abundances of PNMA1 which is absent in the protein content of healthy cells [92], it does suggest that HERV elements are participating in the intricacies cellular biology than previously considered. We emphasize that future studies which investigate the translational efficiency and stability of HERV proteins, and whether pre- or post-translational modifications contributing to their clearance go awry in diseases associated with HERV protein abundance, are of the utmost importance, and continued characterization of the ERT will provide valuable insight into the mysterious mechanisms by which ancient retroviral genes underlie cellular processes as potentially viable and unstudied protein coding genes. These results also suggest reassessment of previous nomenclature that, while lowly abundant in the translatome, might have considered HERVs to be non-coding genes.

## Methods

### Data and code availability

All original code utilized for this study can be found at https://github.com/nixonlab/te_riboseq_atlas. The code for quantifying HERV-provirus aligning reads was adapted from the previously developed hervQuant pipeline [29] which can be found at https://unclineberger.org/vincentlab/resources/. Post hoc visualization of HERV provirus loci was performed with Integrated Genomics Viewer (IGV) [93] desktop application available at https://software.broadinstitute.org/software/igv/. Scatter plots and heatmaps were generated with GraphPad Prism version 9.3.1 available at https://www.graphpad.com/scientific-software/prism/. Biplots displaying PCA differentiation of samples were generated using PCATools available at https://github.com/kevinblighe/PCAtools.

### Quantification of HERV provirus aligned reads from RiboSeq datasets

Quantification of HERVs from the RiboSeq atlas [27] was accomplished using modified methods for the hervQuant pipeline [29]. Briefly, an annotated reference was generated using full-length HERV provirus sequences within hg19 [30]. FASTQ files from were first filtered to remove rRNA reads with Ribodetector [94]. FASTQ reads were then filtered to retain only sequences between 25 and 35 bps in length using SeqKit [95]. Next, known tRNA and rRNA sequences were removed using Bowtie2 v2.5.1 [96]. Post hoc analysis in IGV v2.12.3 [93] demonstrated 5 highly abundant sequences within HERV proviruses that possess high-homology to common RNA contaminants of RiboSeq data [28, 97] based on query searches with BLAST [98] and RNAcentral [99]. These 5 sequences were manually added to the tRNA and rRNA annotation before reanalysis. The final FASTA file of contaminant RNAs removed can be found at https://github.com/nixonlab/te_riboseq_atlas/blob/main/custom_databases/tRNA_rRNA_hg19_ND.fa. Filtered FASTQ files were then aligned to the HERV reference annotation using STAR v2.7.9a [100] (multimaps ≤3 and mismatches ≤1). Next, SAM file outputs were filtered to generate BAM files containing only HERV aligned reads with SAMtools v1.14 [101] before quantification with Salmon v0.8.2 [102] (quant mode − 1 a). For quality assurance, SAMtools v1.14 [101] sorted BAM files merged per tissue type were visualized in IGV v2.12.3 [93]. HERV_1613, HERV_2322, HERV_2740, HERV_4231, HERV_4596, and HERV_5896 were removed from analyses due to RNA contaminant alignment. For RPM abundances, all samples were normalized to filtered reads (Table S2). HERV superfamily annotations were gathered from the supplemental information provided by the original description of hervQuant pipeline [29] and HERV annotations [30]. PCA was performed using PCAtools v2.6.0 on DESEQ2 v1.34.0 [103] transformed objects from raw count matrices. Characterization of translated ORFs was performed using BLAST [98], clustal omega [104], and ORFFinder [105] tools. Statistical analysis was performed using GraphPad Prism v9.3.1. Degree of significance was demonstrated using the following key: *p < 0.05, **p < 0.01, ***p < 0.001, ****p < 0.0001.

### Supplementary Information

**Additional file 1: Supplemental Table 1.**

**Additional file 2: Supplemental Table 2.**

**Additional file 3: Supplemental Table 3.**

**Additional file 4: Supplemental Figures.**

## Data Availability

The datasets analyzed during the current study are available in the Sequence Read Archive (SRA), https://www.ncbi.nlm.nih.gov/bioproject/PRJNA756018. All data was collected from previously published works [27, 106] and can be accessed through the SRA Run Selector under the BioProject number “PRJNA756018”.
