# Supplementary figures and images for: Ribosomal profiling of human endogenous retroviruses in healthy tissues
Source: BMC Genomics. 2024 Jan 2;25:5. doi: 10.1186/s12864-023-09909-x (PMC10759522; doi:10.1186/s12864-023-09909-x)

## Slide 1
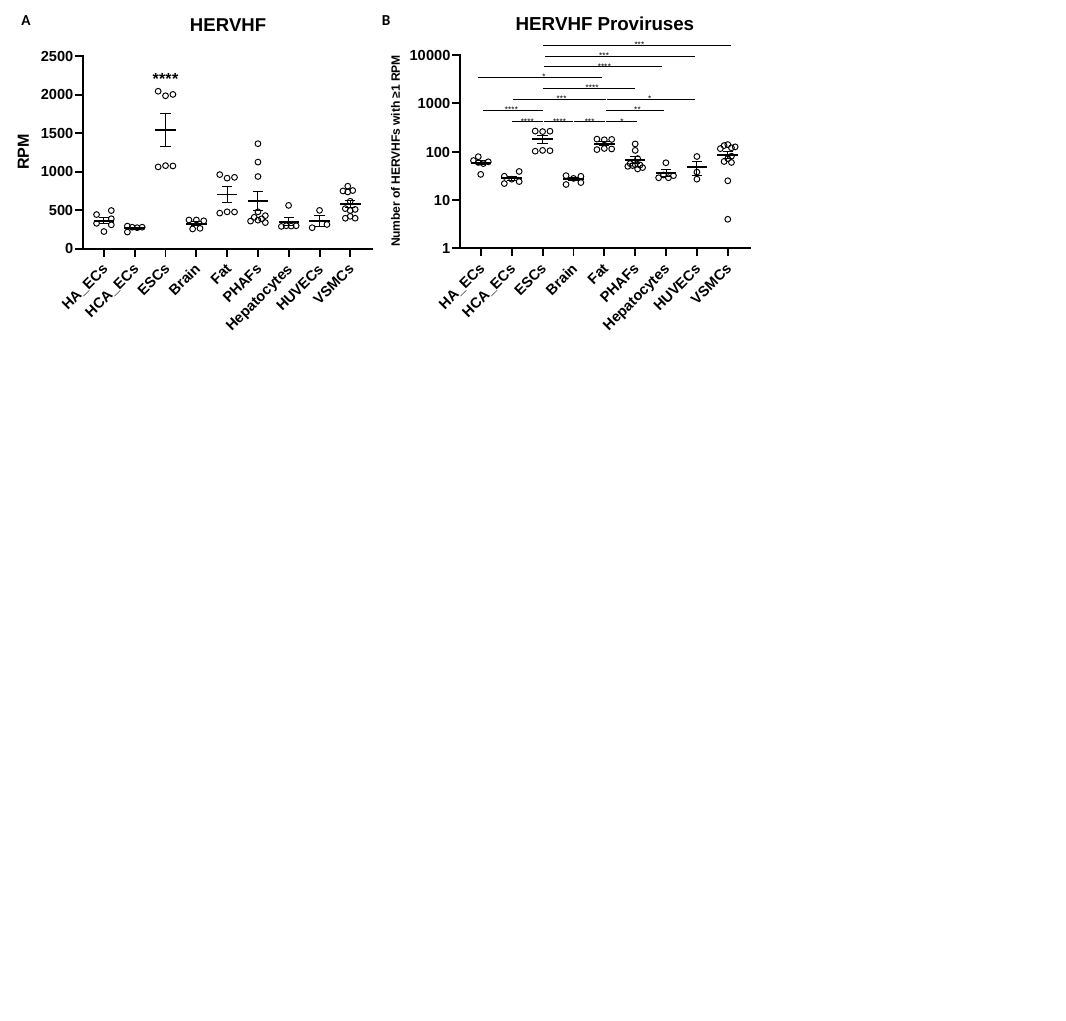

A
B

## Slide 2
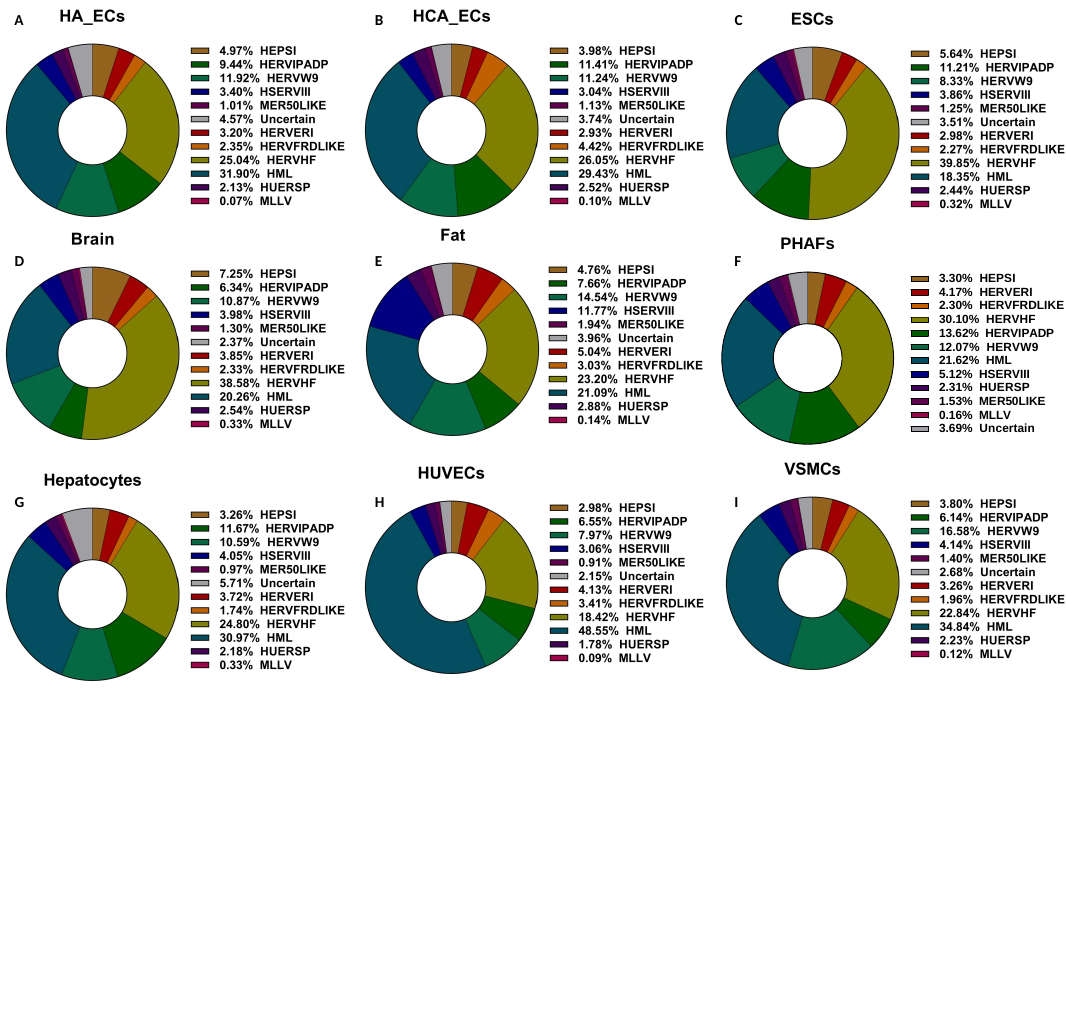

A
B
C
D
E
F
G
H
I

## Slide 3
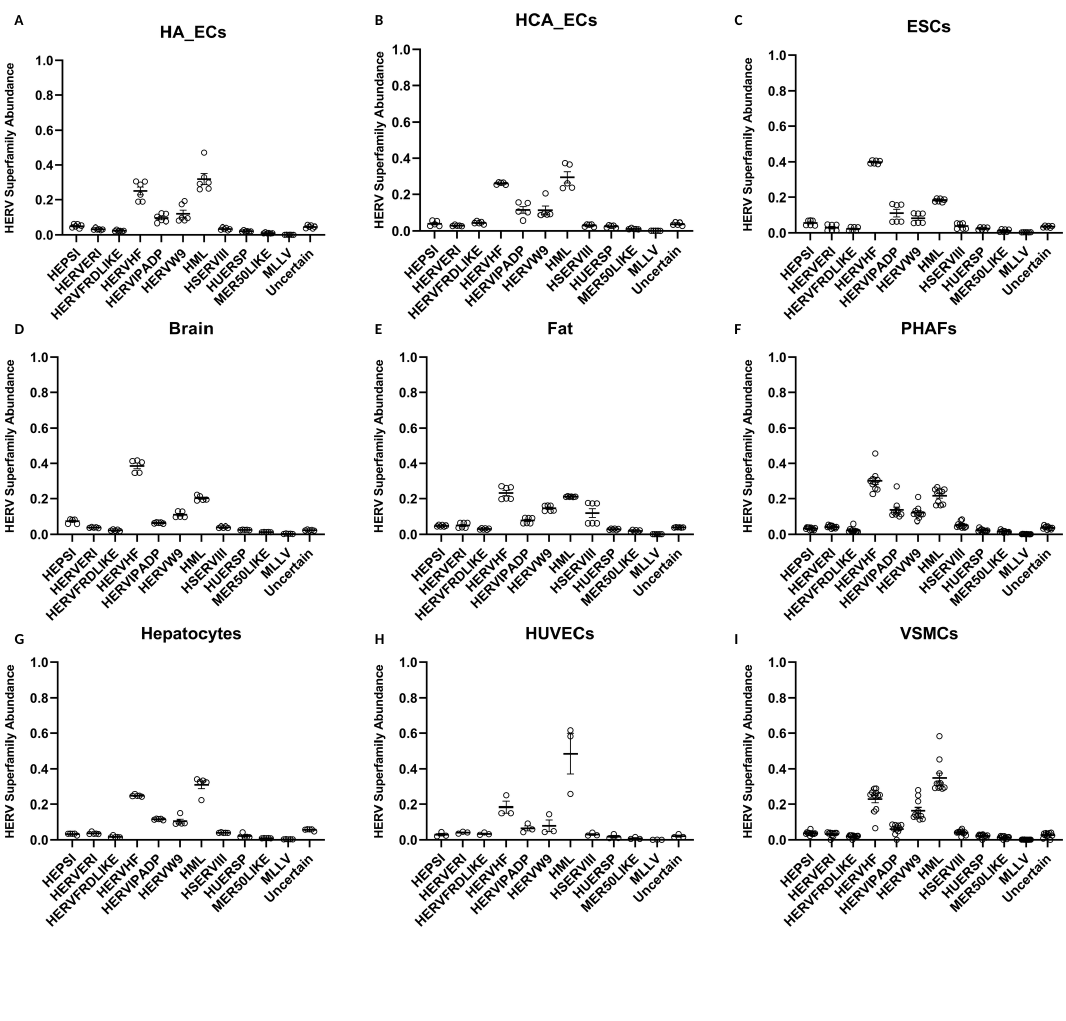

A
B
C
D
E
F
G
H
I

## Slide 4
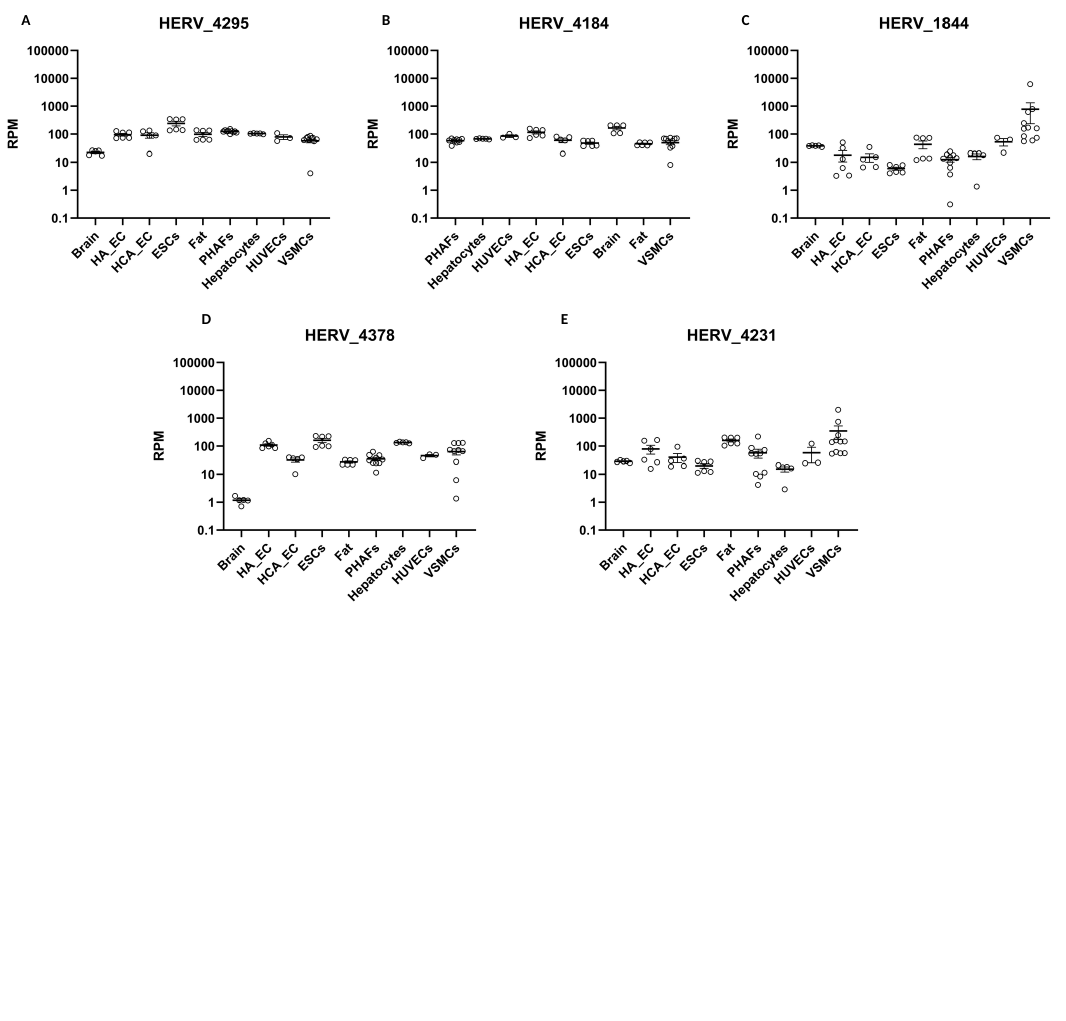

A
B
C
D
E

## Slide 5
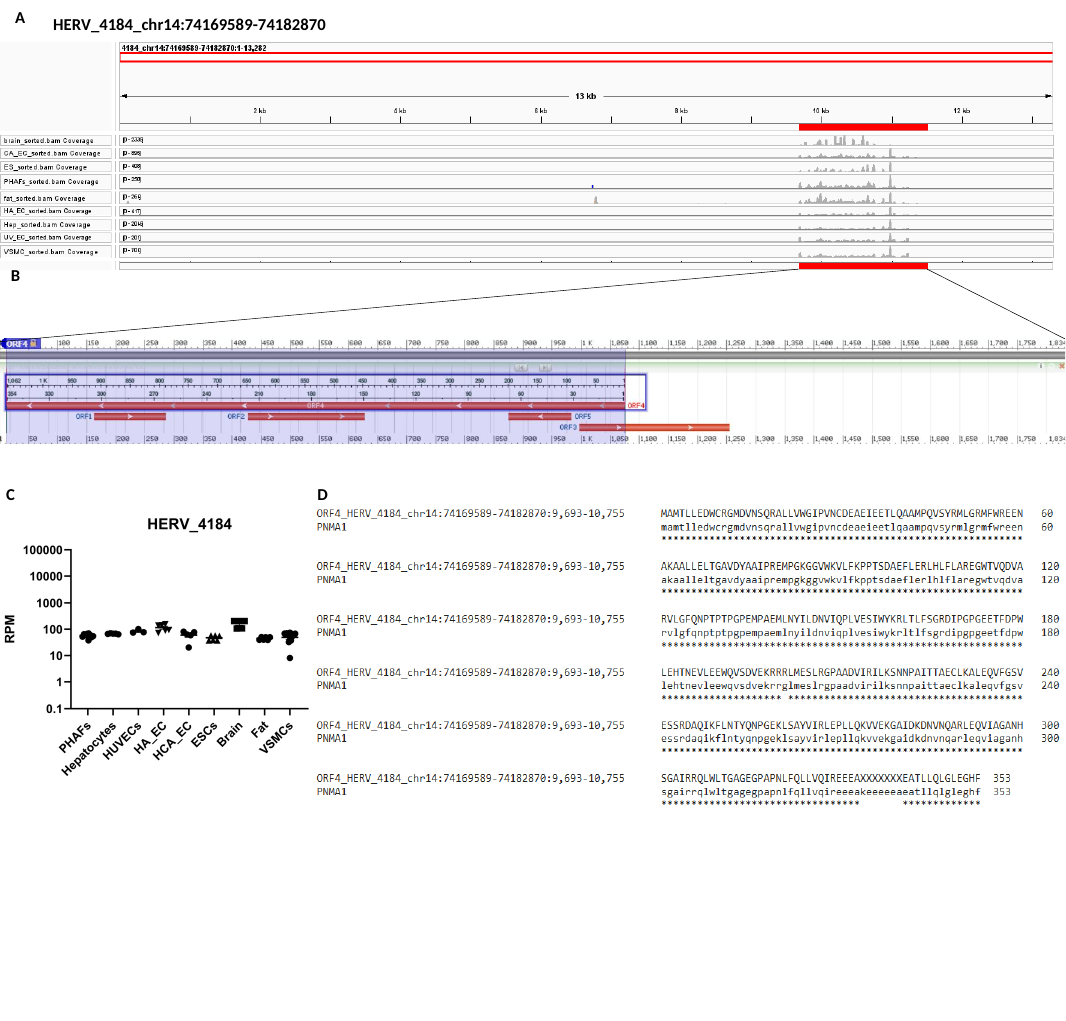

A
HERV_4184_chr14:74169589-74182870
B
C
D

Supplement: Supplementary file 4 — Additional file 4: Supplemental Figures. [file 12864_2023_9909_MOESM4_ESM.pptx]
